# Science, innovation and society: what we need to prepare for the health challenges of the twenty-first century?

**DOI:** 10.1093/inthealth/ihz047

**Published:** 2019-01-01

**Authors:** Jeremy Farrar

**Affiliations:** Wellcome Trust, Gibbs Building, 215 Euston Road, London NW1 2BE, UK

**Keywords:** collaboration, health care, science, innovation and society, research funding

A little over 30 y ago, human immunodeficiency virus (HIV) was first identified as the cause of acquired immune deficiency syndrome (AIDS). Within a decade the nature of the infection, the pathogenesis and a series of combination therapies had been developed that transformed a deadly infection into a challenging but manageable condition. The challenge was then to ensure that everyone who needed these lifesaving therapies had access to them. With more than 20 million people still without access to antiretroviral drugs, that remains a real challenge, but the progress in science and innovation has been truly remarkable.^1^

The start of the HIV pandemic in the mid 1980s was all the more concerning because in the last few decades of the twentieth century infectious diseases around the world were in retreat. Smallpox had been eradicated, terrifying illnesses like polio were starting to be eliminated and common childhood infections of the past, like measles, were increasingly under control through vaccination programmes while antibiotics remained broadly effective and multiple resistance had yet to emerge.

In the first decade of this century, in support of an energised World Health Organization (WHO) and with strong public support, agencies such as the Global Alliance for Vaccine Initiative (GAVI), Global Fund to Fight AIDS, TB and Malaria (Global Fund) and the Joint United Nations Programme on HIV and AIDS (UNAIDS) were established to finance and bring focus through public–private partnership models to support science and innovation and ensure equitable access to essential public health interventions within countries. By creating financial incentives, advance market commitments, guarantees and volume of sales to develop new vaccines, GAVI was able to transform the global research and development for vaccines and ensure those vaccines could reach those who needed them most. Similarly, the Global Fund, UNAIDS and other such partnerships pooled resources from public, private and philanthropic sectors to take on some of the great challenges of our time and by doing so achieved more than any one sector could achieve on its own.

Many others such partnerships followed, accompanied by increasing public and political support for the power of science to transform lives and the commitment to ensure those benefits were available to the maximum number of people globally, independent of their ability to pay. The first draft of the human genome was published in 2001 and through the work of many international organisations, including the US National Institutes of Health, the Wellcome Trust and laboratories in China, France, Germany and Japan, was made publicly available to the world, marking a high point in international scientific collaboration and a clear commitment to sharing and open, safe access to critical scientific knowledge for the benefit of everyone. Almost 20 y on, there is almost no part of public health and medical research that is not touched by the knowledge gained from the Human Genome Project. It has led to advances in technology and data science beyond human health that have transformed almost all areas of biology and will continue to do so throughout this century.

In 2003 the world was reminded of the power of infectious diseases and the impact of globalisation when an epidemic of a novel lung infection, severe acute respiratory syndrome (SARS), spread from southern China to Hong Kong and thereafter to many other countries, including those in Southeast Asia and Canada. SARS was caused by a previously unknown virus the SARS–coronavirus (SARS-CoV), a zoonotic virus that was eventually traced back through an intermediary of civet cats from bats in Yunnan province.^2^ Between November 2002 and July 2013 there were >8000 known cases and almost 800 deaths across 37 countries, with an estimated cost of approximately US$60 billion. Since 2004 there have been a series of major national, regional and global epidemics, including SARS, avian flu, Nipah, Ebola, Zika, yellow fever, non-polio enteroviruses and Middle East respiratory syndrome (MERS). Each demonstrates that we remain vulnerable to endemic and emerging infectious diseases. In low- and middle-income countries, where many infections are endemic, the double burden of infectious diseases and increasing burden of non-communicable diseases pose an enormous challenge to the resilience and adaptability of vulnerable health care systems.

Yet the first decade of this century was a period of real optimism. The advances in prevention and care for people with HIV/AIDS showed what science could achieve when allied to support innovation and when society is engaged and central to the advances. It was an enlightened scientific era, with a clear sense of shared values that enjoyed strong societal support and commitment. There was clear political commitment to multilateral agencies, the United Nations, the World Health Organisation and others as the essential international architecture that brought all the countries of the world together to share challenges and ensure, as far as possible, equitable solutions. In parallel, several major pharmaceutical companies engaged with the global health community, providing donated efficacious products for a group of what have been called neglected tropical diseases, addressing conditions such as leprosy, onchocerciasis, filariasis, trachoma and a range of helminth infections. These donations are valued at some US$2–3 billion annually.^3^

And these commitments changed the world. Between 2000 and 2010 there was a 60% reduction in the number of people dying of malaria. As well as the millions of lives saved, hundreds of millions of people were protected from infection. This was achieved by the introduction at scale of long-lasting insecticide-impregnated nets (LLINs) and artemisinin-based drug therapies, with some 70% of the reduced mortality being attributed to net interventions that were introduced after years of research showing what a difference they could make.^4^ By 2010, there was major progress against many of the world’s most feared infectious diseases by applying advances in science to major public health challenges. This occurred not only in infectious diseases, but almost every area of medicine saw advances, from prevention and treatment of cancer to improving outcomes for patients with heart attacks and diabetes. With these scientific advances, life expectancy increased globally.

This could and should have been the springboard to enhanced health for everyone everywhere, as science advanced with clear public and political support for ensuring the benefits of these advances in improving lives for all. And yet, at this moment of profound optimism, there were in retrospect dark clouds developing. Some of them were inevitable, some of them not, but for many we were complacent. And these will impact on some of the great health challenges of the twenty-first century. They must be addressed with renewed commitment, public support that must not be taken for granted, investment in science and innovation, pooling of resources and an unambiguous commitment to multilateralism, common public goods and equitable access to the benefits of science. These dark clouds, about which we were complacent, are multiple and diffuse, but all have the capacity to roll back the advances in global health made over of the last few decades.

In 1998 a paper was published that claimed a link between the mumps, measles and rubella (MMR) vaccine and autism. Despite being shown to be fraudulent and subsequently retracted, this paper gave rise to the antivaccine movement and has been picked up by anti-establishment politicians around the world.^5^ With the reduction in the uptake of the MMR vaccine there was a 300% increase in the number of cases of measles globally in 2018–2019, with major epidemics on all continents. The antivaccine movement threatens the incredible advances against a range of infectious diseases that were deadly just one generation ago—including measles, polio, rubella and tetanus—but also threatens the opportunity to consign other infectious (and related) diseases to the history books—human papilloma virus and cervical cancer, hepatitis and liver cancer, pneumonia and meningitis (*Haemophilus* influenza and *Streptococcus* pneumonia).

The changing environment driven by climate and ecological change and urbanisation is increasing the frequency and spread of emerging and re-emerging infections. Since the turn of the century we have seen epidemics of novel pathogens—SARS, MERS, strains of influenza (H5N1, H7N9 and others)—and the re-emergence or changing epidemiology of others (Ebola, Zika and non-polio virus enteroviruses). Many of these are zoonotic, and as the pattern of animal–human interactions changes with changing habitats, and consequently reduced biodiversity, it is clear we will see more of these infections. Others are spread by mosquitoes and other vectors, which are exquisitely sensitive to climate change and urbanisation. The mosquito *Aedes aegypti*, which carries dengue, yellow fever, Zika, chikungunya and other viruses, is spreading beyond its traditional tropical belt as climate change increases humidity and temperatures in a much-extended geography and large, densely populated cities provide the ideal environment for the mosquito to thrive. In parallel, the highly adaptable and aggressive Asian tiger mosquito, *Aedes albopictus*, is spreading into new regions, including Italy and France, posing a similar threat given its capacity to transmit several of the same viruses as *A. aegypti*. Many of the above health challenges are exacerbated by the increased frequency of climate-induced natural disasters for which many countries are inadequately prepared. Climate-related changes have been implicated in the ability of *Bulinus* snails to transmit *Schistosoma haematobium* in Corsica.^6^

The inextricable increase in drug resistance also threatens much of the public health progress of the last few decades. The whole of modern medicine, from infection, to routine surgery, to cancer therapy, to looking after patients in critical care units, to those with diabetes or immunodeficiencies, depends on the ability to prevent and treat infections. Drug resistance is now common and growing across all classes of antimicrobials, from antibiotics to antivirals, antifungals and antiparasitics. Resistance is also growing at an alarming rate to insecticides used to impregnate bed nets—an intervention that has been pivotal in the fight against malaria and *Aedes* populations worldwide.^7^ We may soon be, or already are, in a world in which antiretroviral drugs do not work for HIV, artemisinin combinations do not work for malaria, there is no effective drug to treat multidrug-resistant TB and where the risks of infection make it impossible to perform routine surgery.

With progress over the last 50 y against infections and the changing demographics of aging populations, as well as changing diet and exercise, the nature of global health is changing. Cardiovascular disease, cancer, obesity, diabetes and mental health are now the major causes of morbidity and mortality in every country in the world. Scientific advances have, can and will transform these public health issues, but there is a danger that the advances will be not be accessible, even if affordable, by the vast majority of people globally who need them, and in some areas there has been little or no progress over the last few decades. In addition, the delay in achieving registration and WHO approval often delays the implementation of interventions. Mental health is one such area with inadequate progress, whether in prevention and treatment of young people who suffer from mental health issues or in senior people with dementia. These are pressing global crisis that require sustained investment and understanding.^8^

At the heart of many of these issues is the challenge of restructuring often fragile health systems to cope with the changing nature of health and disease. Health systems set up to deal with acute infections inevitably struggle as the pattern of illness shifts to chronic conditions and where prevention is the only economically viable way to address them at a national level. Unlike vaccines, there is no relatively simple technological fix. At a time of increasingly short-term political thinking, it is essential to make the case for sustained investment in public health and health systems appropriate for the changing demographics we will face globally in the twenty-first century. And such investment will not only allow for enhanced health in the face of the rise of non-communicable diseases, it is also the bedrock of efforts to prevent and respond to endemic infectious diseases, providing the capacity to deal with the inevitable epidemics that do so much to destroy societies. It is in all of our interests, in both poor and rich countries, to support universal health coverage and argue for its implementation as a core principle of the United Nations Sustainable Development Goals in reducing poverty and inequity and supporting human rights.

So the dawn of the third decade of the twenty-first century looks very different from a world just 10 y ago. With increasing nationalism and a retreat from a sense of common public good with shared challenges, opportunities and benefits, the antivaccine movements and climate change deniers, fanned by anti-establishment short-term politicians with limited sympathy or understanding of science, climate, conflict (often climate induced) and environmental change, increasing impacts on global health and rapidly changing demographics all challenge the optimism of the first decade of the twenty-first century.

But we are not passive observers of history. We can change the world, and we can and will if we act now. The key is to bring together three elements that when combined enhance each other and ensure that our collective ingenuity benefits the maximum number of people—that is, the combination of science, innovation and society. And the challenges we face in the twenty-first century require integration of all three; perhaps it is time to embrace the lessons of the Rennassiance.^9^

Drug resistance is inevitable, as pathogens are constantly evolving, finding ways to evade our immune systems and our drugs. Infections have always emerged and will continue to—the Black Death and the pandemic flu of 1918 are vivid examples. Science and innovation can develop the next generation of drugs, vaccines and other interventions. However, without an appreciation in society of the value of these interventions, no amount of science can ensure we have what is needed to underpin modern medicine or preventive health care policies. Without publicly supported regulation to control use while ensuring access and robust incentives to address market failures and ensure new science comes into public health within an acceptable time frame to ensure an impact, we will lose these remarkable drugs now and for future generations.

Preparing for the next outbreak means being prepared for the unknown. But we are changing too, and Ebola showed us that a virus does not have to mutate to present a different challenge.^10^ The world’s increasing population, urbanisation, ecological change, loss of biodiversity, freer and faster movement of people, mistrust and conflict are the factors that made it easier for a virus that might have been restricted to an isolated small community in 1976 to spread around a whole region in 2016 causing more than 11 000 deaths. We are so interconnected now that an outbreak in one part of the world is immediately a concern everywhere, with the potential to affect all of us. That shared risk should lead us to share access to the best science and innovation to ensure everyone everywhere who needs it has access to the vaccines, drugs and public health services that science is able to develop.

Climate and environmental change is changing the nature of public health today. They are not just idle threats for some future century, their impact is now. Science and innovation can help provide many of the solutions. However, society must understand and embrace the changes that will be needed and the scientific world needs to reach out and involve communities and become more politically engaged and astute. We must also appreciate that we need to offer hope and that a glorious scientific advance that is not embraced by society will only have limited benefit. Populist political movements, antivaccine advocates, climate change denial and a growing cynicism towards experts and evidence have made these arguments harder, but not impossible. We have to understand the cynicism and get smarter about how we make the case for shared purpose. Shouting louder does not work.

And these same factors are also advantageous. Researchers can pool resources, collaborate across international borders and share data and ideas instantly. Enlightened policymakers can make decisions based on evidence from anywhere in the world. An innovation in India can be applied in Europe. And just as science crosses geographical boundaries, we can break down artificial boundaries between research disciplines. To improve health, we need social sciences to understand the cultures within which we are operating as well as the biology to understand disease and the innovation to develop potential solutions.

At the heart of the Wellcome Trust’s approach to improving health is advancing ideas. We do that by supporting thousands of researchers and other creative people in science, in innovation and in society, wherever they are. But as an international funder, we can do more than fund people and teams with great ideas. We can use our experience, expertise and authority to influence the contexts in which ideas progress and to make sure they can generate the greatest possible benefit. International collaboration and partnerships are critical, a centre of gravity where the issues are at their most acute, as well as multilateral organisations that bring the world together to share the challenges, opportunities and benefits. Bringing science, innovation and society together gives the greatest chance to improve the health of everyone. All our health depends on it.
